# Right atrial tumor revealing intravascular leiomyomatosis: about a case and literature review

**DOI:** 10.1093/jscr/rjae171

**Published:** 2024-03-17

**Authors:** Nourrelhouda Bahlouli, Fatima Chait, Khadija Laasri, Nazik Allali, Latifa Chat, Siham El Haddad

**Affiliations:** Pediatric Teaching Hospital, Radiology department, Mohammed V University, Rabat 6527, Morocco; Pediatric Teaching Hospital, Radiology department, Mohammed V University, Rabat 6527, Morocco; Pediatric Teaching Hospital, Radiology department, Mohammed V University, Rabat 6527, Morocco; Pediatric Teaching Hospital, Radiology department, Mohammed V University, Rabat 6527, Morocco; Pediatric Teaching Hospital, Radiology department, Mohammed V University, Rabat 6527, Morocco; Pediatric Teaching Hospital, Radiology department, Mohammed V University, Rabat 6527, Morocco

**Keywords:** uterine leiomyomatosis, right orielette mass, intravascular metastasis, inferior vena cava

## Abstract

Intravascular leiomyomatosis (IVL) is a very rare extension of uterine leiomyoma through the pelvic vessels. It is a benign pathology with malignant potential with a possibility of intra-cardiac extension and metastases (cerebral, pulmonary, lymph node); early diagnosis is very difficult. Prognosis depends on involvement of the inferior vena cava and extension to the right cavities. We report a case of complications of IVL, precisely the extension in the right atrium, in a 49-year-old woman. The objective of our work is to highlight the importance of imaging in the diagnosis of the vascular extension of leimyomatosis.

## Introduction

Intravenous leiomyomatosis (IVL) is characterized by the infiltration of venous channels by a histologically benign tumor composed of smooth muscles, originating either from the vessel wall or from a uterine leiomyoma. It should not be confused with benign metastasizing leiomyoma (BML), which involves the presence of leiomyomas in a distant organ other than the uterus. Intracardiac extension is rarely reported. The average age is 45 years. The pathophysiology remains unknown.

### Case report

This is the case of a 49-year-old woman who is being treated for high blood pressure by amlodipine, 5 mg. The patient experienced difficulty in breathing. A trans-thoracic echocardiogram was performed, revealing a tumor in the right atrium. She underwent surgery in October 2020, during which a complete resection of the mass was performed along with tricuspid annuloplasty.

**Figure 1:**
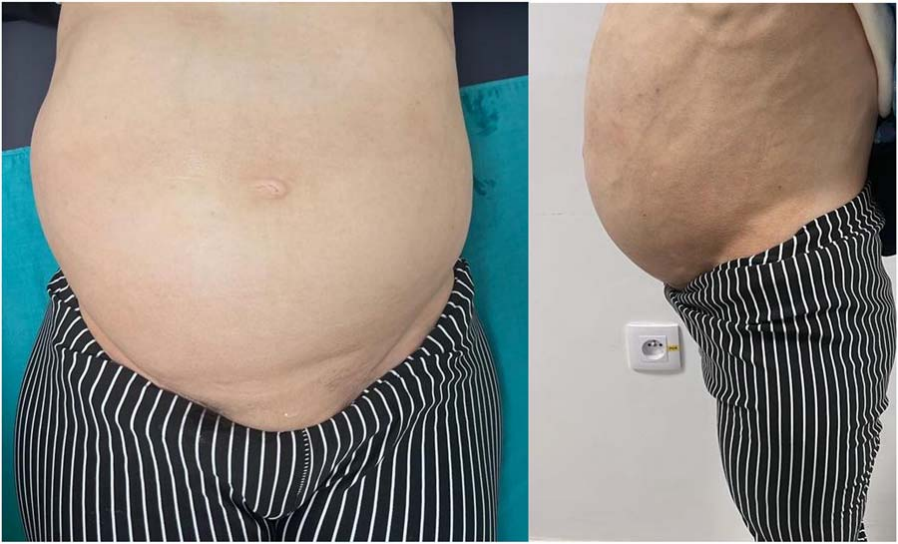
Real images of the substantial abdominal-pelvic swelling, both front and side views.

A few months later, the patient developed an abdominal and pelvic mass (Fig. 1) An ultrasound was performed, revealing a uterus with multiple fibroids. At the same time, due to worsening shortness of breath, a trans-thoracic echocardiogram was conducted, which revealed a recurrence of the mass in the right atrium, adherent to the wall and extending into the inferior vena cava, measuring 12 cm. The patient underwent a second surgery to manage this recurrence.

**Figure 2:**
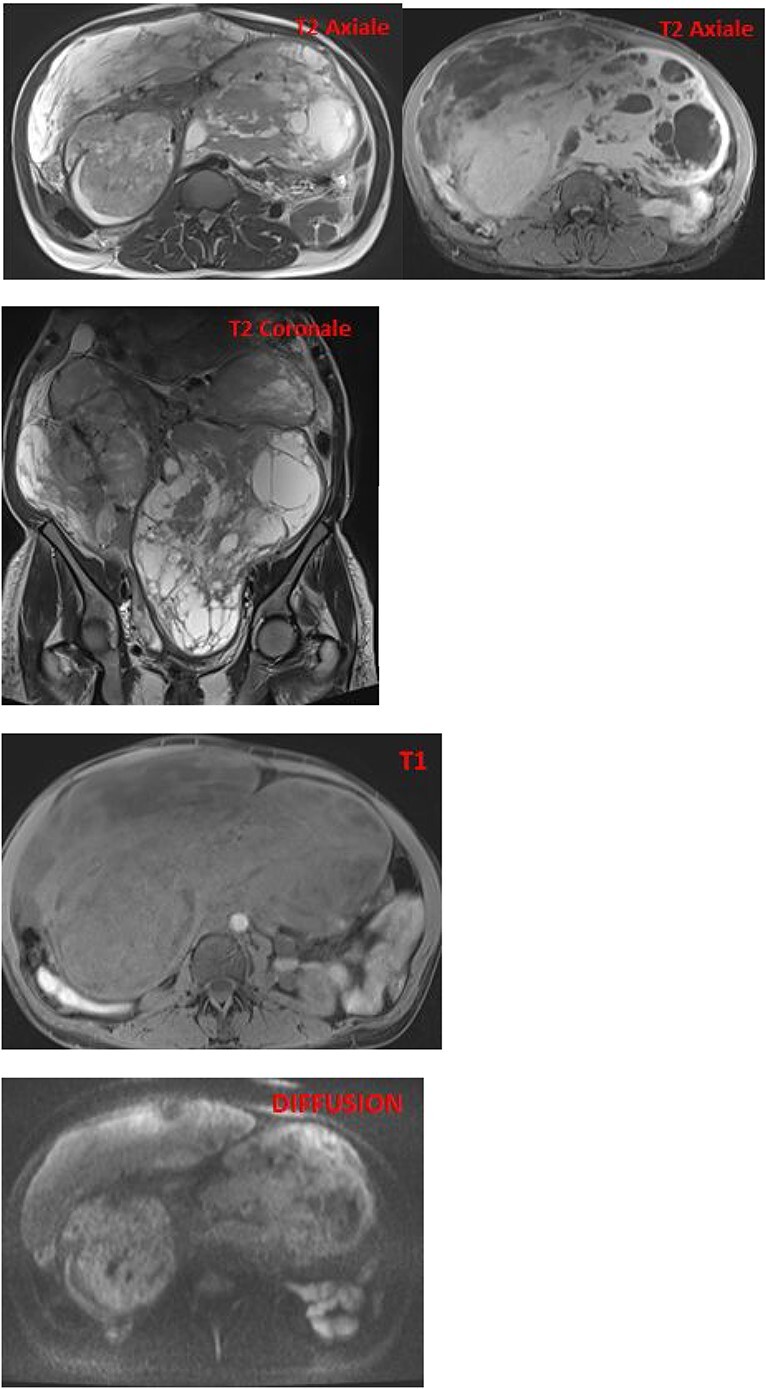
MRI images reveal an enlarged uterus with multiple myometrial formations exhibiting intermediate signal on T1 and T2, encompassing regions of hyperintensity on T2 and hypointensity on T1, enhanced homogeneously compared with the myometrium and without diffusion restriction, the most characteristic ones are classified as FIGO 6, FIGO 7, and FIGO 2–5, suggesting uterine leiomyomatosis with cystic degeneration.

**Figure 3:**
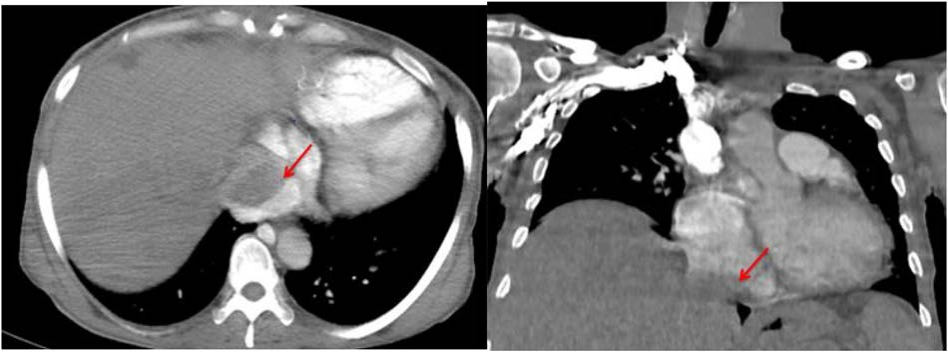
Angio-CT thoracic images reveal the presence of a mass in the inferior vena cava extending into the right atrium (arrow).

**Figure 4:**
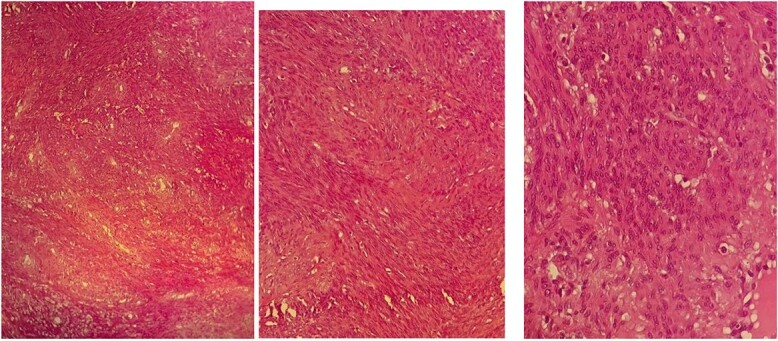
Pathological images of HE-stained slides with three levels of magnification: (a) ×200, (b) ×400, (c) ×1000; proliferation of spindle-shaped cells without cyto-nuclear atypia, very low mitotic activity, and negative tumor necrosis; the appearance suggests benignity.

**Figure 5:**
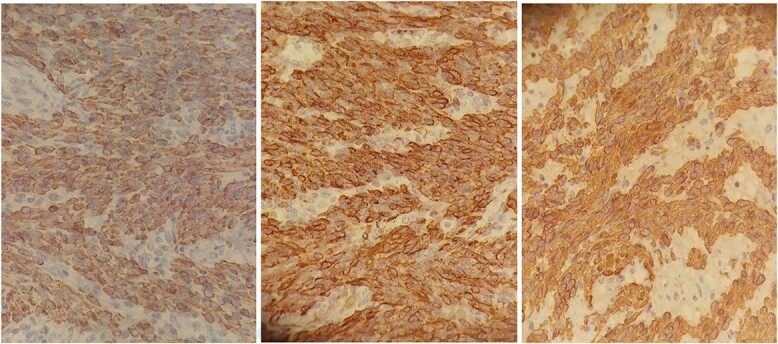
Anatomo-pathological images with immunohistochemical staining: smooth muscle actin antibody +, Desmin antibody +, Codexin antibody + suggestive of smooth muscle differentiation resembling myometrial cells.

The pathological and immunohistochemical examination suggested a smooth muscle mesenchymal tumor without signs of malignancy, raising the possibility of disseminated intravascular leiomyomatosis or a BML (Figs 4 and 5). The thoracic computed tomography (CT) indicated the presence of a mass in the inferior vena cava extending into the right atrium. Magnetic resonance imaging (MRI) confirmed uterine leiomyomatosis (Fig. 3). This explains the origin of the mass in the right atrium.

Exploring the inferior vena cava at its intra-abdominal portion was difficult because it was compressed by the uterine mass (Fig. 2). Following discussions and collaboration among vascular surgery, gynecology, anesthesiology, cardiothoracic surgery, and intensive care, the diagnosis of intravascular leiomyomatosis was confirmed.

## Discussion

Uterine leiomyomatosis is a benign and rare tumor. It involves the presence of leiomyomas outside the genital tract. This condition includes IVL, BML, disseminated peritoneal leiomyomatosis, and hereditary leiomyomatosis [1]. It typically occurs in the perimenopausal stage, often in individuals with a history of hysterectomy and/or uterine leiomyomas. It can lead to various complications, such as extension into the parametrium, ovarian veins, inferior vena cava, and further into the right atrium and cardiac chambers. This extension can be life-threatening [2]. The first case was described in the literature in 1896 [3]. Intracardiac extension was first described in 1907 by Durck [2].

IVL is the extension of smooth muscle cells through the veins. There are two proposed pathophysiological hypotheses. The first corresponds to the venous extension of a leiomyoma, while the second relates to a tumor developed from the walls of the veins [4]. Intravascular extension occurs in 75% of cases in the parametrial vessels and in 25% in the uterine and hypogastric veins [3]. Rarely, it can extend into the common iliac, renal, and adrenal veins, and exceptionally (10%–40% of cases) [5, 6], or into the right heart chambers or the pulmonary artery.

Clinical symptoms may include cardiac signs such as syncope, cardiac myxoma-like symptoms, right heart failure, Budd-Chiari syndrome, systemic embolism, and pulmonary embolism [7]. Recurrent ascites and sudden death are also potential clinical manifestations [3]. Additionally, it may present with a sensation of abdominal heaviness, increased abdominal volume, lower limb edema, and can even be discovered postoperatively.

Cardiac ultrasound can raise suspicion of IVL by detecting a mass in the right atrium and the inferior vena cava.

Contrast-enhanced ultrasound helps to fill the gaps in conventional ultrasound. It reveals slow blood flow within the vessels, aiding in the diagnosis of IVL intra vascular leimyomatosis [8]. Currently, a study involving 14 patients has revealed the existence of two types of IVL: solid and cystic masses [9].

CT and MRI are crucial for determining the size and shape of the uterus (Figs 2 and 3), confirming the myometrial origin of the mass, and specifying the extent of vascular involvement. This classification distinguishes four grades [1]:

- Grade 1: invasion of uterine vessels by the tumor without extra-pelvic involvement.
- Grade 2: extension into the abdominal cavity without affecting the renal vein.
- Grade 3: involvement of the renal vein, inferior vena cava, and the right atrium.
- Grade 4: involvement of pulmonary arteries and/or the presence of pulmonary metastases.

On MRI, the appearance of endovascular material depends on the quantity of smooth muscle cells and fibrous tissue. Generally, it exhibits low to intermediate signal on T1 and low signal on T2 [6] (Fig. 2).

The differential diagnosis with gynecological causes includes uterine leiomyoma, leiomyosarcoma, adenomyosis, or myofibroma [10]. Regarding cardiac involvement, it can be distinguished from atrial myxoma, thrombotic material, and endovascular tumor extension from renal cell carcinoma or hepatocellular carcinoma. In the case of inferior vena cava involvement, it should be differentiated from leiomyosarcoma of the inferior vena cava.

The treatment involves total hysterectomy with the removal of the tumor and bilateral adnexectomy. Ovariectomy is crucial in the management because it is a hormonally sensitive tumor (estrogen-dependent). According to some authors, a two-stage surgery is preferred for large tumors [11]. Thrombectomy of the inferior vena cava and iliac vein is performed in cases of intravascular involvement. Thrombectomy of the right atrium and inferior vena cava with extracorporeal circulation may also be necessary [12]. It is important to note that extracting the tumor from the right atrium alone carries a high risk of pulmonary embolism or inferior vena cava tear, as the origin is in the pelvic veins [1].

In the presence of residual cells, treatment with tamoxifen is recommended [13].

The risk of recurrence is 30%. Therefore, it is essential to provide monitoring through abdominal-pelvic ultrasound and inferior vena cava ultrasound, along with a thoraco-abdomino-pelvic CT scan.

## Conclusion

The incidence of IVL is increasing. It is crucial to consider this condition when encountering a tumor in the right atrium of a perimenopausal woman. Imaging, especially MRI, is highly valuable for exploring the para-uterine vessels. A comprehensive assessment is essential before any surgical intervention to guide the treatment approach.

A thorough assessment is crucial before any surgical procedure to guide the surgical treatment.

## Conflict of interest statement

The authors declare that they have no known competing financial interests or personal relationships that could have appeared to inf luence the work reported in this paper.

## Funding

This research received no specific grant from any funding agency in the public, commercial, or not-for-profit sectors.
